# Effectiveness and safety of rhIGF1 therapy in patients with or without Laron syndrome

**DOI:** 10.1530/EJE-20-0325

**Published:** 2020-11-17

**Authors:** Peter Bang, Joachim Woelfle, Valerie Perrot, Caroline Sert, Michel Polak

**Affiliations:** 1 Division of Pediatrics, Department of Biomedical and Clinical Sciences, Faculty of Health Sciences, Linköping University, Linköping, Sweden; 2 Children’s Hospital, University of Erlangen, Erlangen, Germany; 3 Ipsen Pharma, Boulogne-Billancourt, France; 4 Department of Paediatric Endocrinology, Gynaecology, and Diabetology, AP-HP, Necker-Enfants Malades University Hospital, IMAGINE Institute, University of Paris, Paris, France

## Abstract

**Objective:**

The European Increlex^®^ Growth Forum Database Registry monitors the effectiveness and safety of recombinant human insulin-like growth factor-1 (rhIGF1; mecasermin, Increlex^®^) therapy in patients with severe primary IGF1 deficiency (SPIGFD). We present data from patients with and without a reported genetic diagnosis of Laron syndrome (LS).

**Design:**

Ongoing, open-label, observational registry (NCT00903110).

**Methods:**

Children and adolescents receiving rhIGF1 therapy from 10 European countries were enrolled in 2008–2017 (*n* = 242). The treatment-naïve/prepubertal (NPP) cohort (*n* = 138) was divided into subgroups based on reported genetic diagnosis of LS (*n* = 21) or non-LS (*n* = 117). Multivariate analysis of the NPP-non-LS subgroup was conducted to identify factors predictive of growth response (first-year-height standard deviation score (SDS) gain ≥ 0.3). Assessments included change in height and weight over 5 years and adverse events (AEs).

**Results:**

Height SDS gain from baseline was greater in the NPP-LS than the NPP-non-LS subgroup after 1 years’ treatment (*P <* 0.05). In the NPP-non-LS subgroup, 56% were responders; young age at baseline was a positive independent predictive factor (*P* < 0.001). NPP-non-LS-responders and the NPP-LS subgroup had a similar mean age (6.07 years vs 7.00 years) at baseline and height SDS gain in year 1 (0.64 vs 0.70), although NPP-non-LS-responders were taller (*P* < 0.001) at baseline. BMI SDS changes did not differ across subgroups. Treatment-emergent AEs were experienced by 65.3% of patients; hypoglycaemia was most common.

**Conclusions:**

In most NPP children with SPIGFD, with or without LS, rhIGF1 therapy promotes linear growth. The safety profile was consistent with previous studies.

## Introduction

Severe primary insulin-like growth factor deficiency (SPIGFD) is associated with postnatal growth failure (1). It is characterised by very low levels of insulin-like growth factor-1 (IGF1) and other growth hormone (GH)-regulated proteins, despite normal or elevated GH secretion (2, 3). In patients with Laron syndrome (LS), SPIGFD results from a mutation in the GH receptor gene, causing GH insensitivity and severe-to-extreme short stature (–4 to –10 height standard deviation score (SDS), increasing with age) (2, 3). In addition to proportionate short stature, phenotypic characteristics of LS include frontal bossing, hypoplastic midface, central obesity, small genitalia and delayed puberty (4, 5). Other genetic causes of SPIGFD, include STAT5b and acid-labile subunit (ALS) mutations (6, 7). However, most patients with severe growth stunting and biochemical characteristics of SPIGFD cannot be assigned a causative genetic diagnosis (8).

Recombinant human IGF1 (rhIGF1; mecasermin, Increlex^®^; Ipsen Pharma, Boulogne-Billancourt, France) therapy stimulates linear growth in children with SPIGFD (9, 10) and improves adult height (11). In Europe, rhIGF1 therapy was approved for treating growth failure in children with SPIGFD in 2007 (12). The European Increlex^®^ Growth Forum Database (Eu-IGFD) Registry was set up to collect long-term safety and effectiveness data from clinical practice (13).

In clinical trials, growth response to rhIGF1 therapy was shown to be dose-dependent (9). However, data collected from the Eu-IGFD Registry up to September 2013 demonstrated that rhIGF1 therapy effectiveness varied between subgroups (13). In patients who were treatment-naïve/prepubertal, a younger age and lower baseline height SDS, characteristics of the LS subgroup included in the analysis, were predictors of a greater change in height SDS in year 1 of rhIGF1 therapy (13). Further analyses to determine and verify predictive response factors for patients with SPIGFD without LS would help to inform clinicians’ treatment decisions.

The objective of the presented primary analyses was to evaluate the effectiveness and safety of up to 5 years of rhIGF1 therapy in children with SPIGFD, with or without a reported genetic diagnosis of LS. However, as most participants in the Eu-IGFD Registry do not present with LS, it would be beneficial to better characterise clinical characteristics of these patients. Therefore, the secondary analyses aimed to better describe baseline characteristics of patients without LS in whom rhIGF1 therapy was most effective, in order to support clinicians in their treatment decisions.

## Methods

### Study design

The Eu-IGFD Registry is an ongoing, open-label, observational study monitoring rhIGF1 therapy use in children with growth failure due to SPIGFD (NCT00903110). Enrolment criteria, study procedures and assessments have been reported previously (13). The EuIGFD Registry, started in 2008, includes children and adolescents with growth failure from 10 European countries, receiving rhIGF1 therapy, who provided informed consent or assent, as appropriate. Diagnosis of SPIGFD was judged by the reporting physician and was not possible to reassess for reasons previously detailed (13). Baseline was the visit closest to rhIGF1 therapy start. The cut-off date for analyses was 10 May 2017 (ENCEPP/SDPP/7708).

The Eu-IGFD Registry is conducted in compliance with independent ethics committees/institutional review boards (except the UK, where the ethical review is not required for this registry type), informed consent regulations, the Declaration of Helsinki, International Conference on Harmonization, and Good Epidemiological Practice Guidelines.

### Patient subgroups

**Figure 1:**
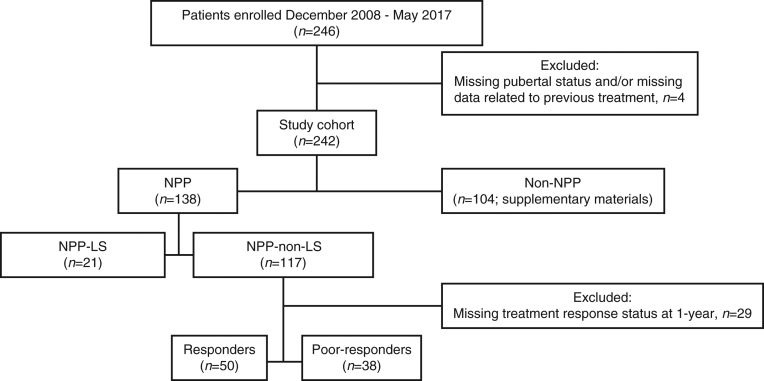
Study disposition and patient subgroup distribution. Responders were defined as patients with a change in height SDS in year 1 of ≥0.3; poor-responders were defined as patients with a change in height SDS in year 1 of <0.3. In the registry and safety populations, three patients from the NPP-non-LS subgroup and one patient from the non-NPP cohort were excluded as no follow-up visits were performed. Non-NPP, not treatment naïve and/or pubertal; NPP, treatment-naïve and prepubertal; LS, Laron syndrome.

Patients were divided into two cohorts (Fig. 1), that is, treatment-naïve and prepubertal (NPP), and not treatment-naïve and/or pubertal (non-NPP). Patients receiving recombinant human growth hormone (rhGH), rhIGF1 therapy and steroids were classed as non-NPP. Prepubertal was defined as Tanner stage 1 genital or breast development for boys and girls, respectively. Within each cohort, patients were split into two subgroups based on whether they had genetically verified LS (LS or non-LS). One patient reported with LS was diagnosed based on a typical phenotype, which was not genetically verified. For all other patients with LS, a GH receptor deletion or mutation was confirmed by genetic analysis.

### Safety analyses

All adverse events (AEs), serious AEs, and treatment-emergent AEs (TEAEs) were analysed. Targeted TEAEs (13) were defined as AEs occurring while treatment was ongoing, which were either frequently or historically associated with rhIGF1 therapy (regardless of whether they were considered drug related).

### Statistical analysis

The enrolled population (baseline characteristics data) comprised patients who had complete, clinical research associate-monitored baseline data (13); the registry population (effectiveness data) comprised patients who received ≥1 dose of rhIGF1 therapy and completed ≥1 follow-up visit; and the safety population (safety data) comprised patients who received ≥1 dose of rhIGF1 therapy and who attended ≥1 follow-up visit, or for whom there were post-study treatment safety data.

Calculations of weight, BMI, height SDS, and height velocity were performed as reported previously (13). Results from descriptive analyses are presented as mean (s.d. (95% CI)) and/or median (first and third quartile: Q1; Q3). Categorical data were analysed using Chi-square or Fisher’s exact test and quantitative data were analysed using ANOVA or Wilcoxon test. Statistical analyses were exploratory so no adjustment for multiplicity was performed.

### Univariate and multivariate analyses

Logistic regression analysis was used to identify factors predictive of growth response at year 1 by comparing NPP-non-LS-responders with poor-responders (gain in height of ≥0.3 SDS or <0.3 SDS after 1 year of treatment, respectively; Fig. 1). The cut-off was based on the previously reported mean change in height of the registry population at year 1, and suggested cut-offs in patients with GH insensitivity (13, 14, 15). A univariate analysis used the following potential factors: sex, mid-parental adult height, birth height; and the following baseline parameters: age, height SDS, weight SDS, IGF1 level, IGF binding protein-3 and rhIGF1 therapy initial dose and dose during year 1. Potentially important factors identified from the univariate analysis (significant at the 20% level) were included in the final multivariate model.

## Results

Overall, 246 patients enrolled in the Eu-IGFD Registry from December 2008 to May 2017, and 138 were included in the NPP cohort analyses; with LS (*n* = 21), or without LS (*n* = 117; Fig. 1). The heterogeneity of the non-NPP cohort (*n* = 104) did not allow for meaningful efficacy reporting. The non-NPP cohort baseline characteristics, puberty and previous treatment status, rhIGF1 therapy dosing and changes in height SDS, height velocity, BMI SDS and weight SDS are provided in Supplementary materials (Supplementary Tables 1, 2, 3, 4, 5 and 6, see section on supplementary materials given at the end of this article). All baseline and efficacy data reported below refer to the NPP cohort.

### Primary analyses: effectiveness of rhIGF1 therapy in NPP patients with or without LS

Baseline characteristics of LS and non-LS subgroups are presented in Table 1. The LS subgroup was significantly younger (*P* = 0.006), had a more severe short stature (*P* < 0.001), lower IGF1 levels (*P* = 0.007), and higher GH secretion (*P* = 0.014) compared with the non-LS subgroup at baseline. History of spontaneous hypoglycaemia before rhIGF1 therapy was more frequently observed in those with LS compared with those without LS: 4 (19%) and 3 (2.6%) patients, respectively (*P* = 0.011). The LS subgroup had a significantly higher rhIGF1 therapy starting dose compared with the non-LS subgroup (median (Q1; Q3): 40 (40; 40) vs 40 (20; 40) µg/kg BID; *P* = 0.013), but no significant difference in dose was observed from year 1 to year 5 after dose escalation. Duration of treatment was not significantly different between those with and without LS (median (Q1; Q3): 4.97 (2.09; 6.35) vs 3.90 (1.98; 5.28) years).

**Table 1:** Treatment-naïve/prepubertal (NPP) patient characteristics at baseline in patients with or without LS (Enrolled population).

Characteristics	NPP-LS (*n* = 21)					NPP-non-LS (*n* = 117)					*P* value^a^
	*n*^b^	*n* (%)	Mean (s.d.)	95% CI	Median (Q1; Q3)	*n*^b^	*n* (%)	Mean (s.d.)	95% CI	Median (Q1; Q3)	
Females	21	9 (42.9)		24.5; 63.5		117	46 (39.3)		30.9; 48.4		0.76^c^
Age at first injection, years	21		6.07 (3.49)	4.49; 7.66	6.11 (2.82; 8.30)	117		8.44 (3.45)	7.81; 9.07	8.23 (5.70; 11.21)	0.006^f^
Height, SDS	16		−5.62 (1.95)	−6.66; −4.58	−5.88 (−6.89; −4.22)	109		−3.46 (1.05)	−3.66; −3.26	−3.26 (−3.82; −2.88)	<0.001^f^
Height velocity, cm/year	7		5.67 (1.10)	4.66; 6.69	5.64 (5.12; 6.24)	63		4.74 (1.77)	4.29; 5.19	4.91 (3.95; 5.71)	0.174^f^
Weight, SDS	17		−4.63 (1.35)	−5.32; −3.93	−4.61 (−5.44; −3.83)	110		−3.04 (1.12)	−3.26; −2.83	−3.07 (−3.57; −2.32)	<0.001^f^
BMI, SDS	16		−0.24 (1.30)	−0.94; 0.45	−0.19 (−0.92; 0.29)	98		−0.80 (1.34)	−1.07; −0.53	−0.76 (−1.70; 0.02)	0.126^e^
Mother’s height, cm	18		156.0 (7.4)	152.3; 159.7	155.3 (151.9; 160.0)	108		157.8 (7.2)	156.4; 159.2	158.0 (153.0; 162.7)	–
Father’s height, cm	18		168.5 (7.6)	164.7; 172.3	169.2 (164.5; 174.8)	107		172.6 (8.1)	171.0; 174.1	172.0 (168.0; 178.0)	–
IGF1, ng/mL	9		39.37 (16.25)	26.89; 51.86	37.00 (25.00; 38.93)	103		88.30 (67.89)	75.04; 101.57	70.5 (39.00; 119.00)	0.007^f^
Peak stimulated GH level, ng/mL	13		35.50 (20.53)	23.10; 47.91	33.30 (25.00; 38.3)	84		24.61 (24.87)	19.22; 30.01	15.10 (11.25; 26.10)	0.014^f^
Primary diagnosis: SPIGFD^†^	21	21 (100)		84.5; 100.0		117	106 (90.6)		83.9; 94.7		–
History of hypoglycaemia	21	4 (19.0)		7.7; 40.0		117	3 (2.6)		0.9; 7.3		0.011^d^

^†^Including LS, as reported by the investigator; ^a^NPP-non-LS vs NPP-LS; ^b^number of patients with available data; ^c^Chi-square test; ^d^Fisher’s test; ^e^ANOVA; ^f^Wilcoxon test.

BMI, body mass index; GH, growth hormone; IGF1; insulin-like growth factor-1; SDS, standard deviation score; SPIGFD, severe primary IGF1 deficiency.

**Figure 2:**
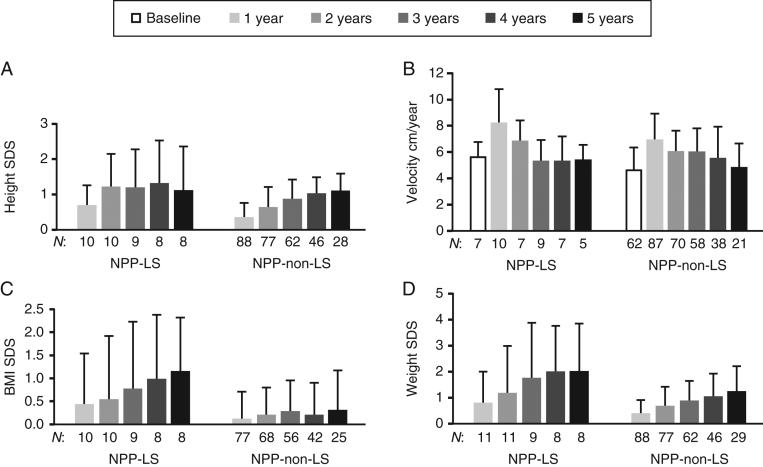
Effect of rhIGF1 therapy on height SDS (A), height velocity (B), BMI SDS (C), and weight SDS (D) in treatment-naïve/prepubertal (NPP) patients with or without LS (registry population). Data are mean (s.d.). *n*, number of patients with available data at each time point; LS, Laron syndrome; SDS, standard deviation score.

While height SDS gain in the LS subgroup was observed during the first 2 years of treatment, the non-LS subgroup appeared to gain height SDS during subsequent years (Fig. 2, Table 2 and Supplementary Table 4). Height SDS gain was significantly greater in the LS subgroup compared with the non-LS subgroup at year 1 (*P* = 0.019) and year 2 (*P* = 0.044). However, in years 3, 4 and 5, height SDS gain was not significantly different between the subgroups. There was also an apparent greater increase in BMI SDS and weight SDS from baseline in the LS subgroup vs the non-LS subgroup; however, weight SDS was only significantly greater in the LS subgroup at year 4 (*P* = 0.022) (Fig. 2 and Supplementary Tables 5, 6).

**Table 2:** Effect of rhIGF1 therapy on height SDS in the treatment-naïve/prepubertal (NPP) subgroups with or without LS (registry population). Mean (s.d./95% CI) are not reported if the *n* is < 5. Responders were defined as patients with a change in height SDS in year 1 of ≥ 0.3. Poor-responders were defined as patients with a change in height SDS in year 1 of < 0.3.

	NPP-LS (*n* = 21)				NPP-non-LS (*n* = 114)				*P* value^a^	NPP-non-LS-responders (*n* = 50)				NPP-non-LS-poor-responders (*n* = 38)				*P* value^b^
			Change from baseline				Change from baseline					Change from baseline				Change from baseline		
	*n*	Mean (s.d.)	*n*	Mean (95% CI)	*n*	Mean (s.d.)	*n*	Mean (95% CI)		*n*	Mean (s.d.)	*n*	Mean (95% CI)	*n*	Mean (s.d.)	*n*	Mean (95% CI)	
1 year	15	–4.68 (1.83)	10	0.70 (0.29; 1.10)	95	–3.07 (1.05)	88	0.37 (0.28; 0.45)	0.019	50	–2.85 (1.11)	50	0.64 (0.57; 0.71)	38	–3.44 (0.96)	38	0.01 (–0.06; 0.08)	<0.001
2 years	14	–4.26 (1.85)	10	1.23 (0.57; 1.88)	81	–2.83 (1.09)	77	0.64 (0.51; 0.77)	0.044	40	–2.54 (1.07)	40	0.91 (0.77; 1.05)	29	–3.28 (1.10)	29	0.20 (0.06; 0.34)	<0.001
3 years	11	–3.85 (2.11)	9	1.20 (0.36; 2.03)	67	–2.51 (1.15)	62	0.88 (0.73; 1.02)	0.173	33	–2.25 (1.06)	33	1.15 (0.98; 1.32)	23	–2.87 (1.33)	23	0.55 (0.31; 0.78)	<0.001
4 years	10	–3.83 (2.14)	8	1.33 (0.33; 2.33)	49	–2.25 (1.08)	46	1.03 (0.89; 1.17)	0.211	25	–2.15 (1.11)	25	1.21 (1.03; 1.40)	13	–2.52 (0.90)	13	0.76 (0.54; 0.99)	0.004
5 years	10	–3.99 (2.34)	8	1.12 (0.08; 2.15)	32	–1.93 (1.03)	28	1.11 (0.92; 1.30)	0.983	20	–1.92 (0.96)	20	1.25 (1.06; 1.45)	5	–2.33 (0.26)	5	0.73 (0.15; 1.31)	0.045

^a^NPP-non-LS vs NPP-LS mean height SDS. ^b^NPP-non-LSe-responders vs NPP-non-LS-poor-responders mean height SDS.

*n*, number of patients with available data at each time point. LS, Laron syndrome; **SDS, standard deviation score.

### Predictive factors of rhIGF1 therapy response in the NPP non-LS-subgroups

To characterise factors predictive of growth response at year 1 in patients without LS, the NPP patients were divided into responders and poor-responders. Just over half of patients were responders (56.8%; Fig. 1). The univariate analysis demonstrated that in NPP patients without LS, age, baseline IGF1, and mid-parental adult height were potentially important factors predictive of growth response at year 1. However, the final multivariate analysis only identified a statistically significant correlation between the change in height SDS during year 1 and age at baseline (odds ratio: 0.75 (95% CI 0.65; 0.87); *P* < 0.001), with younger patients being better responders to rhIGF1 therapy than older patients. Responders were significantly younger than poor-responders (by approximately 4.5 years (median); *P* < 0.001; Table 3); however, all other baseline characteristics, including height SDS, were similar between subgroups.

**Table 3:** Treatment-naïve/prepubertal (NPP) patient characteristics at baseline in responders and poor-responders without LS (enrolled population). Responders were defined as patients with change in height SDS in year 1 of ≥0.3; poor-responders were defined as patients with change in height SDS in year 1 of <0.3.

Characteristics	NPP-non LS poor responder (*n* = 38)					NPP-non-LS responder (*n* = 50)					*P* value^a^
	*n*^b^	*n* (%)	Mean (s.d.)	95% CI	Median (Q1; Q3)	*n* ^b^	*n* (%)	Mean (s.d.)	95% CI	Median (Q1; Q3)	
Females	38	11 (28.9)		17.0; 44.8		50	20 (40.0)		27.6; 53.8		0.282^c^
Age at first injection, years	38		10.28 (3.53)	9.12; 11.44	10.86 (7.82; 13.47)	50		7.00 (3.11)	6.12; 7.88	6.28 (4.19; 9.33)	<0.001^f^
Height, SDS	38		−3.44 (0.90)	−3.74; −3.15	−3.28 (−3.74; −3.03)	50		−3.49 (1.15)	−3.81; −3.16	−3.27 (−3.99; −2.84)	0.930^f^
Height velocity, cm/year	18		4.19 (1.98)	3.20; 5.17	3.98 (2.74; 5.57)	35		4.99 (1.66)	4.42; 5.56	5.19 (4.31; 5.77)	0.117^f^
Weight, SDS	37		−3.14 (0.89)	−3.43; −2.84	−3.09 (−3.52; −2.68)	50		−3.07 (1.02)	−3.36; −2.78	−3.04 (−3.52; −2.57)	0.758^e^
BMI, SDS	33		−1.11 (1.30)	−1.57; −0.65	−1.27 (−1.90; −0.11)	44		−0.67 (1.28)	−1.06; −0.28	−0.73 (−1.26; 0.19)	0.138^e^
Mother’s height, cm	36		158.4 (7.9)	155.7; 161.1	158.5 (153.3; 165.0)	46		158.6 (6.8)	156.5; 160.6	158.0 (155.0; 162.0)	–
Father’s height, cm	36		174.8 (7.6)	172.3; 177.4	174.5 (170.0; 180.0)	46		171.4 (8.1)	169.0; 173.8	172.0 (167.0; 176.0)	–
IGF1, ng/mL	35		110.32 (77.11)	83.83; 136.80	91.00 (61.00; 139.00)	42		86.97 (69.95)	65.17; 108.76	68.25 (31.30; 110.00)	0.069^f^
Peak stimulated GH level, ng/mL	27		22.51 (19.08)	14.96; 30.05	16.01 (11.50; 24.40)	35		24.76 (27.15)	15.43; 34.08	13.60 (10.90; 26.10)	0.865^f^
Primary diagnosis: SPIGFD^†^	38	35 (92.1)		79.2; 97.3		50	43 (86.0)		73.8; 93.0		–
History of hypoglycaemia	38	0		0; 9.2		50	2 (4.0)		1.1; 13.5		0.504^d^

^†^Including LS, as reported by the investigator. ^a^NPP-non-LS poor-responder vs NPP-non-LS responder; ^b^number of patients with available data. ^c^Chi-square test; ^d^Fisher’s test; ^e^ANOVA; ^f^Wilcoxon test.

BMI, body mass index; GH, growth hormone; IGF1, insulin-like growth factor-1; LS, Laron syndrome; SDS, standard deviation score; SPIGFD, severe primary IGF1 deficiency.

### Secondary analyses: NPP-non-LS-responders, poor-responders and NPP-LS subgroup comparison

The similarities and differences between the two NPP subgroups that responded best to rhIGF1 therapy were analysed: patients with LS and responders without LS. At baseline, responders without LS and patients with LS were of similar age. However, patients with LS had a significantly lower height SDS, weight SDS, and serum IGF1 level, and higher peak stimulated GH levels, compared with responders without LS (*P* < 0.001, *P* < 0.001, *P* = 0.027 and *P* = 0.025). Responders started on a slightly lower rhIGF1 therapy starting dose compared to patients with LS (*P* = 0.011) but with no difference observed from year 1 to year 5. Responders were treated for a similar duration compared to patients with LS (*P* = 0.990).

During year 1, responders without LS had a similar mean (S.D.) change in height SDS vs patients with LS (0.64 (0.26) vs 0.70 (0.56); *P* = 0.835). This was also reflected in the mean first year height velocities (Supplementary Table 4); mean height SDS gain was greatest in year 1 and continued to steadily increase in years 2–3 then remained stable in years 3–5 (Table 2). Other than BMI SDS at year 4 (*P* = 0.032), changes in BMI SDS and weight SDS from baseline were not significantly different between subgroups (Supplementary Tables 5 and 6).

**Figure 3:**
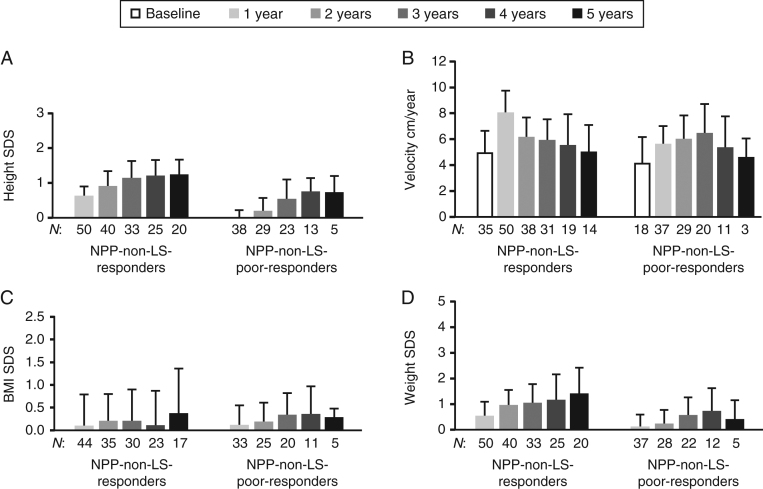
Effect of rhIGF1 therapy on height SDS (A), height velocity (B), BMI SDS (C), and weight SDS (D) in treatment-naïve/prepubertal (NPP) responders and poor-responders without LS (registry population). Data are mean (s.d.). Responders were defined as patients with a change in height SDS in year 1 of ≥ 0.3. Poor-responders were defined as patients with a change in height SDS in year 1 of < 0.3. *n*, number of patients with available data at each time point. LS, Laron syndrome; non-NPP, not treatment naïve and/or pubertal; SDS, standard deviation score.

Among NPP patients without LS, poor-responders and responders had similar rhIGF1 therapy doses from baseline to year 5; however, duration of treatment was significantly shorter in poor-responders (median: 3.47 vs 4.59 years; *P* = 0.028). There were no significant differences in BMI SDS changes at any time point between responders and poor-responders (Fig. 3). However, corresponding to the increase in height, responders had a significantly greater increase in weight SDS compared with poor-responders each year, except at year 4 (years 1–2: *P* < 0.001; year 3: *P* < 0.017; year 5: *P* = 0.038).

### Safety profile

An overview of TEAEs (including the non-NPP cohort; *n* = 242) is presented in Table 4. Overall, 65.3% of patients experienced a TEAE, 20.2% experienced a serious TEAE and 5.4% had a TEAE that led to treatment withdrawal. The most frequently reported TEAEs were hypoglycaemia (*n* = 93), headache (*n* = 41), lipohypertrophy (*n* = 35) and middle ear infection (*n* = 26). The most common serious TEAEs were hypoglycaemia (*n* = 6 (2.5%)), followed by adenoidal and tonsillar hypertrophy (both: *n* = 4 (1.7%)). There were 3 benign and 1 malignant neoplasm TEAEs. The benign neoplasia events were mild in nature: dysplastic naevus occurred in 1 patient (0.4%) (NPP-non-LS-poor-responder; no pre-existing conditions) and melanocytic naevus occurred in 2 patients (0.8%) (NPP-non-LS-responder and NPP-non-LS-poor-responder; pre-existing asthma and no pre-existing conditions, respectively). The malignant event was fatal myelodysplastic syndrome which occurred in 1 patient (non-NPP–non-LS; multiple pre-existing conditions, including thrombocytopenia), as reported by Bang *et al*.** (13). One other TEAE was fatal: a complication of a bone marrow transplant in a patient classified as NPP-responder without LS.

**Table 4:** Overall summary of treatment-emergent adverse events (safety population; *n* = 242).

**Classification**	*n***(%)**
Patients with ≥1 TEAE	158 (65.3)
Patients with ≥1 targeted TEAE	119 (49.2)
Patients with ≥1 serious TEAE	49 (20.2)
Patients with ≥1 serious related TEAE	26 (10.7)
Patients with ≥1 serious targeted TEAE	15 (6.2)
Patients with ≥1 TEAE leading to treatment withdrawal	13 (5.4)
Patients with a fatal TEAE	2 (0.8)

TEAE, treatment-emergent adverse event.

**Figure 4:**
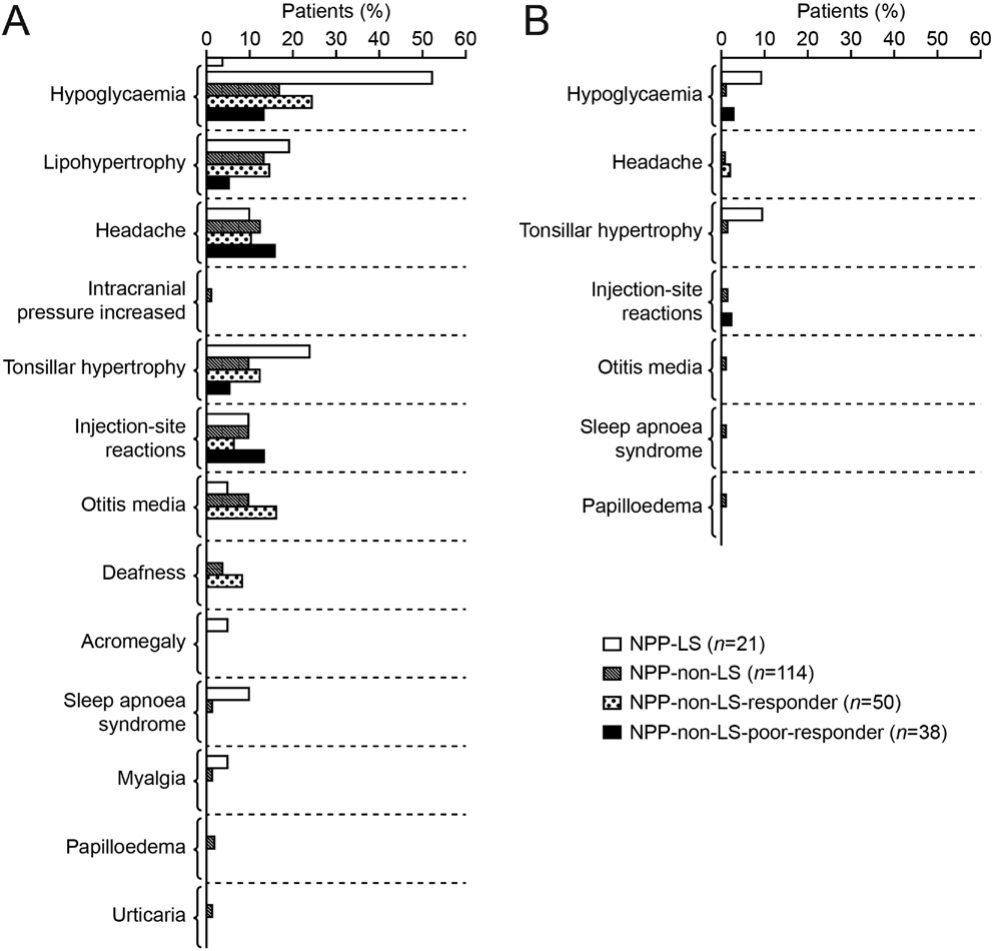
All targeted TEAEs for treatment-naïve/prepubertal (NPP) patient subgroups (A) total, (B) serious (safety population). All reported targeted TEAEs and serious targeted TEAEs are shown. Targeted TEAEs were: headache, chronic middle ear infection, papilledema, hypoglycaemia, acromegalic facial changes, oedema, gynaecomastia, hearing loss, intracranial hypertension, lipohypertrophy at injection sites, myalgia, sleep apnoea, tonsillar hypertrophy and cardiomegaly. Hearing impairment was coded as deafness. Responders were defined as patients with a change in height SDS in year 1 of ≥0.3. Poor-responders were defined as patients with a change in height SDS in year 1 of <0.3. LS, Laron syndrome; TEAE, treatment-emergent adverse event.

Within the treatment naïve/prepubertal cohort, there was an apparent higher frequency of targeted TEAEs in patients with LS (71.4%) compared with those without LS (46.5%; responder: 48.0%, poor-responder: 36.8%). The distribution of total and serious targeted TEAEs across the NPP cohort is presented in Fig. 4.

## Discussion

These analyses demonstrate that rhIGF1 therapy promotes linear growth in children with SPIGFD but its effectiveness varies among subgroups of patients. As previously reported (16), NPP patients with LS responded significantly better to rhIGF1 therapy vs NPP patients without LS. However, the severity of GH insensitivity and genetic diagnosis confirming LS were not the only factors to determine a good response to rhIGF1 therapy. Among NPP patients without LS, a majority of younger patients with less severe signs of GH insensitivity responded similarly well to rhIGF1 therapy as the subgroup of NPP patients with LS. This finding was in accordance with our previous observation that young age is a positive predictor of a good height response in patients with SPIGFD, although this analysis included patients with LS (13).

### Primary analyses: effectiveness of rhIGF1 therapy in patients with or without LS

The Eu-IGFD registry has a relatively large enrolment of patients with LS and, to our knowledge, there have been no other real-world studies that compare NPPl patients with LS vs those without LS. As expected, within the NPP cohort, the LS subgroup was significantly younger, had a more severe short stature, lower IGF1 levels and higher GH secretion at baseline compared with those without LS. Also in line with expectations, the LS subgroup responded significantly better to rhIGF1 therapy, in terms of height SDS gain, compared with patients without LS after 1–2 years of treatment. Conversely, in years 3–5, height SDS remained stable in the LS subgroup, whereas height SDS continued to increase in those without LS. However, this finding could be due to patients leaving the study, as patients without LS who ended treatment before year 3 are likely to have been those with the poorest response. Timing of puberty may also impact the interpretation of long-term growth response to rhIGF1 therapy, as patients with LS are reported to have delayed pubertal development (5).

Excessive weight gain has previously been reported in patients with LS and SPIGFD rhIGF1 therapy trials (5, 17), but this may have been due to encouraged snacking to prevent hypoglycaemia (12). Interestingly, a moderate increase in BMI (~1.0 SDS) was observed over 4–5 years of treatment in NPP patients with LS, which is less than reported by Backeljauw *et al*.** (11), but greater than described in a case report of two slightly older patients (18) and greater than responders without LS in this analysis. Further research in this area would be of interest, to show whether the BMI trends observed here are significant in a larger patient population.

### Predictive factors of rhIGF1 therapy response in the NPP-non-LS subgroup

We adopted a cut-off level for the response to treatment (gain in height SDS: 0.3) that could be considered unsatisfactory for most patients with an approved indication for rhGH treatment. As previously argued, rhIGF1 therapy in SPIGFD is unable to compensate for IGF1 independent actions of GH on growth (13). Furthermore, a similar cut-off was suggested for patients with a genetic defect in the IGF1 receptor treated with rhGH, a condition expected to have lower responsiveness than approved GH indications (14, 15).

Importantly, over half of NPP patients without LS were rhIGF1 therapy responders, with similar treatment response to those in a Polish study of patients without a LS phenotype (*n* = 27) (19). However, age at the start of treatment was the only baseline characteristic that predicted a better response to treatment in this population. Responders were approximately 4.5 years younger than poor-responders. Given that those untreated children with SPIGFD experience a decrease in height SDS as they age (20), these data suggest that rhIGF1 therapy initiation is delayed in children with less severe short stature. Furthermore, the limited gain in height SDS over 5 years in poor-responders underlines the importance of evaluating height response and TEAEs after 1 year and considering whether to stop or continue treatment.

### Secondary analysis: NPP-non-LS-responders and NPP-LS subgroup comparison

Interestingly, patients in the NPP subgroup with LS and responders without LS started rhIGF1 therapy at a similar age, despite patients in the LS subgroup having a more severe short stature, lower IGF1 levels and higher GH secretion at baseline. These data suggest that the severity of short stature and clear biochemical abnormalities may not always lead to early treatment initiation in clinical practice. Notably, our data show that responders in the subgroup of NPP patients without LS had a similar initial and long-term mean gain in height SDS as those with LS . Thus, in SPIGFD, a genetic diagnosis is not necessarily required for a clinically significant height response. However, the LS subgroup remained shorter in stature than responders without LS. In this respect, the height response to rhGH therapy in patients with severe GH deficiency is more marked (21, 22).

### Non-NPP subgroups

The heterogeneity of the non-NPP cohort does not allow for meaningful reporting; therefore, we refrain from interpreting the baseline and effectiveness of rhIGF1 therapy data in these patients.

### Safety profile

Overall, safety data were consistent with the known profile of rhIGF1 therapy (9, 13). Hypoglycaemia was the most frequent TEAE and serious TEAE, in agreement with previous reports (9, 13), although hypoglycaemia is known to occur spontaneously in patients with LS (11). Targeted TEAEs were reported in a higher proportion of patients with than without LS.

Given that IGF1 has mitogenic and anti-apoptotic effects (23), rhIGF1 therapy may stimulate the growth of benign and malignant tumours that pre-exist or develop in patients with SPIGFD (12). Furthermore, IGF1 deficiency in untreated LS is thought to decrease the incidence of cancer (5). There have been post-marketing reports of both benign and malignant neoplasms in children and adolescents who have received rhIGF1 therapy; these cases represented a variety of different and rare malignancies (12). Although available data do not allow calculations of relative risk, the current analyses include four benign and one malignant neoplasm TEAEs. In those who receive rhIGF1 therapy for unapproved uses or at above the recommended doses, risk of neoplasia may be higher. Clinicians should be vigilant for potential malignancy symptoms and if neoplasia develops, rhIGF1 therapy should be discontinued definitely and appropriate expert medical care sought. However, the data in this study do not raise any new safety concerns.

### Limitations

Given the observational nature of the Eu-IGFD Registry, inherent limitations exist as reported earlier (13). The disparity in age between subgroups at baseline could be considered a limitation; however, it supported our finding that age can be used to predict response to treatment. As previously reported, due to the number of different IGF1 assays used by the centres, it is not possible to determine how many patients fulfil the European diagnostic criteria of SPIGFD (IGF1 <2.5th percentile) (13). Measurements of IGF1 levels at baseline may have been confounded by the use of local rather than central laboratory analysis, as results obtained from different assays can vary considerably (24). Furthermore, IGF1 SDS was not available, therefore age could have impacted analyses of IGF1 levels. As data were collected from sites across 10 European countries, differences in diagnostic and treatment practices and in reporting standards are expected. International collaboration is needed to ensure a consistent approach to the identification and management of rare growth diseases such as SPIGFD (25). This analysis is limited by the small patient cohort, expected with a rare disease, therefore the statistical analysis should be interpreted with caution. Despite these limitations, the Eu-IGFD Registry provides valuable insights into the real-world effectiveness of rhIGF1 therapy in subgroups of clinical interest.

## Conclusion

Our analyses demonstrate that rhIGF1 therapy effectively promoted linear growth in the majority of NPP children with SPIGFD. Furthermore, young age at treatment initiation was a positive predictive factor of treatment response in NPP patients without LS, underlining the importance of starting treatment early. Moreover, NPP patients with LS and responders without LS had a similar initial and long-term clinically significant mean gain in height SDS, demonstrating a genetic diagnosis confirming LS was not necessarily a prerequisite for the response. The most common TEAE was hypoglycaemia, reported at a rate of 0.1 events and 0.01 serious events per treatment years. The data in this study do not raise any new safety concerns; however, clinicians and patients should remain vigilant for benign or malignant neoplasia development and, if detected, discontinue treatment immediately and seek appropriate expert medical care.

## Supplementary Material

Supplementary materials

Table S1: Puberty and previous treatment status of the non-treatment-naïve/prepubertal subgroups (Enrolled population)

Table S2: Non-treatment naïve/pubertal patient characteristics at baseline, with or without Laron syndrome (Enrolled population)

Table S3. Effect of rhIGF-1 therapy on height SDS in the non-treatment-naïve/prepubertal cohort (Registry population)

Table S4. Effect of rhIGF-1 therapy on height velocity (cm/year; Registry population)

Table S5. Effect of rhIGF-1 therapy on BMI SDS (Registry population)

Table S6. Effect of rhIGF-1 therapy on weight SDS (Registry population)

## Declaration of interest

P B: advisory board/board of directors fees from Ipsen, Lilly; and consulting fees from Ipsen, Sandoz, Pfizer, Lilly, Versatis. J W: advisory board/board of directors fees from Ipsen, Novo Nordisk; corporate-sponsored research fees from Pfizer, Ipsen; and speaker fees from Merck-Serono, Hexal, Pfizer, Novo Nordisk. V P and C S are employees of Ipsen. M P: advisory board/board of directors fees from Ipsen (Increlex registry), Novo Nordisk (Global Norditropin Advisory board), Pfizer, France; corporate-sponsored research fees from Ipsen, Novo Nordisk, Pfizer, Sandoz, Merck, Sanofi; and speaker fees from Novo Nordisk, Ipsen.

## Funding

This study is supported by Ipsen http://dx.doi.org/10.13039/501100014382.

## Medical writing support

The authors thank Cara Valvona, PhD, of Watermeadow Medical, an Ashfield Company, for providing medical writing support, which was sponsored by Ipsen in accordance with Good Publication Practice guidelines.

## Data sharing statement

Where patient data can be anonymised, Ipsen will share all individual participant data that underlie the results reported in this article with qualified researchers who provide a valid research question. Study documents, such as the study protocol and clinical study report, are not always available. Proposals should be submitted to DataSharing@Ipsen.com and will be assessed by a scientific review board. Data are available beginning 6 months and ending 5 years after publication; after this time, only raw data may be available.

## Author contribution statement

P B, J W, V P, C S and M P were involved in substantial contributions to study conception and design, acquisition and interpretation of data, drafting the publication and revising it critically for important intellectual content and final approval of the publication.
