# Supplementary material for: Effectiveness and safety of rhIGF1 therapy in patients with or without Laron syndrome
Source: Eur J Endocrinol. 2020 Nov 17;184(2):267–76. doi: 10.1530/EJE-20-0325 (PMC7849377; doi:10.1530/EJE-20-0325)
Supplement: Supplementary materials [file supplementary_material.pdf]

Supplementary materials

Non-treatment-naïve and/or pubertal patient subgroup analysis

More than half of both non-treatment-naïve/prepubertal subgroups were prepubertal. Although 81% of the non-treatment-naïve/prepubertal Laron syndrome subgroup received rhIGF-1 therapy prior to enrolment in the Eu-IGFD Registry (Table S1), patients had a significantly shorter stature, significantly higher BMI SDS and peak stimulated GH level, and were significantly more likely to have history of hypoglycaemia than the non-treatment-naïve/prepubertal patients without Laron syndrome at baseline (Table S2). The non-treatment-naïve/prepubertal Laron syndrome subgroup was treated with rhIGF-1 therapy for longer (*P*=0.004) and had a higher starting dose compared with the non-treatment-naïve/prepubertal patients without Laron syndrome (median [Q1; Q3]: 90 [40; 100] versus 40 [40; 40] µg/kg BID; *P*=0.001), however, no significant difference in doses were observed from years 1-5 (median: 112-120 µg/kg BID). Changes in height, height velocity (Table S3 and S4), BMI and weight (Table S5 and S6) for the non-treatment-naïve/prepubertal subgroups should be interpreted with caution, due to the heterogeneity of the subgroups (Table S1).

Discussion of the non-treatment-naïve and/or pubertal patient subgroup data

As expected, the non-treatment-naïve/prepubertal Laron syndrome subgroup had higher GH levels than the non-treatment-naïve/prepubertal patients without Laron syndrome at baseline, while the higher mean serum IGF-1 in the non-treatment-naïve/prepubertal Laron syndrome subgroup at baseline was not expected given that levels of IGF-binding protein-3 and ALS are GH-dependent. However, a high percentage of the non-treatment-naïve/prepubertal Laron syndrome subgroup had been previously treated with rhIGF-1 therapy, which may have elevated the baseline IGF-1 levels, depending on how soon treatment occurred prior to sample collection [1].

The heterogeneity of the non- treatment-naïve/prepubertal subgroups does not allow for meaningful reporting, therefore we refrain from interpreting the effectiveness of rhIGF-1 therapy in these patients. The majority of patients in the non-treatment-naïve/prepubertal Laron syndrome subgroup were likely to have had their catch-up growth prior to entering the Eu-IGFD Registry, and therefore were not expected to gain much additional height during pubertal years. This has been observed in patients with GH deficiency receiving GH for many prepubertal years [2] and is also in accordance with the dynamics of catch-up growth in response to rhIGF-1 therapy in patients with Laron syndrome/SPIGFD reported here. Again, these data should be interpreted with caution until adult height data analyses are available.

Eu-IGFD Registry Study Group – active sites

Austria: D. Furthner, G. Hausler, K. Zwiauer; **Belgium**: M.C. Lebrethon, J. de Schepper; **France**: P. Adiceam, C. Braun, B. Cammas, M. Cartigny, C. Cessans*, C. Cessans†, M. Cogne, R. Coutant, M. de Kerdanet, C. Gayet (previously E. Mallet), P. Hassler, M. Houang, A. Lienhardt, A. Linglart, N. Lucidarme, F. M'Bou, H. Malpuech-Rouffet, M. Nicolino, F. Njuieyon, M. Pinget, M. Polak, R. Reynaud, P.F. Souchon, M.T. Tauber, K. Wagner; **Germany:** S. Al Sawaf, M. Bald, S. Bechtold, M. Bettendorf J.-G. Blanke, K. Cordula, D. Dunstheimer, A. Enniger (previously H. Leichter), M. Frühwald, C. Grasemann, K. Hartmann, B. Hauffa, E. Hammer, P.-M. Holterhus, A. Hübner, J. Ittner, C. Jourdan, A. Keller, H.S. Kim-Berger, B. Köster (previously Rosenbaum), J. Krüger (previously Richter-Unruh), P. Kuhnle coinv, C. Land, F. Lorenzen, M. Marx, T. Meissner, K. Mohnike, M. Morlot, H. Müller, P. Müller, C. Ockert, R. Oeverink, K. Palm, C.-J. Partsch, T. Reinehr, T. Rohrer, E. Schönau, E. Schulz, K.

Schwab, G. Simic-Shleicher, B. Tittel, K. Warncke, J. Wölfle; **Italy:** C. Bizzarri, S. Cannavò, M. Cappa, V. Cherubini, O. Cotta, G. Citro, D. Concolino, M-F. Fainza (previously L. Cavallo), G. Festa, D. Fintini, P. Francesco Perri, G. Garbetta, L Guazzarotti, A. Lampis, S. Longhi, E. Marchesi, S. Mauri, M. Maghnie, R. Minelli, E. Miraglia Del Giudice, A. Moscillo, G. Patti, L. Perrone, A. Pilotta, S. Rabbiosi, A. Sinisi, G. Tornese, G. Weber (previously G. Chuimello), S. Zucchini; **Netherlands**: A. Hokken-Koelega, **Poland:** I. Ben-Skowronek, D. Birkholz, A. Bossowski, A. Dudek, M. Hilczer, A. Kedzia, K. Majewska, M. Niedziela, B. Rabska-Pietrzak, K. Stozek, J. Smyczynska, G. Zandwijken; **Spain:** J. Argente, C. Bezanilla, M-F. Borras, M. Caimari, R. Diaz, A. Feliu-Rovira, C. Fernandez, M. Ferrer, E. Gallego, A Gutierrez, F. Hermoso, A. Lechuga-Sancho, C. Luzuriaga, J. Martos, M-F. Moreno-Macian, C. Pavia, P. Prieto, C. Rodriguez, A. Vela, D. Yeste; **Sweden:** P. Bang, A. Holmgren (previously N.-Ö. Nilsson), A. Tidblad; **United Kingdom:** F. Ahmed, L. Denvir, H. Johnstone, T. Mushtaq, L. Patel, C. Peters, R. Ramakrishnan, S. Rose, N. Shaw, H. Storr.

*Catie Cessans; [†]Catherine Cessans

References

1. Ekstrom K, Carlsson-Skwirut C, Ritzen EM & Bang P. Insulin-like growth factor-I and insulin-like growth factor binding protein-3 cotreatment versus insulin-like growth factor-I alone in two brothers with growth hormone insensitivity syndrome: effects on insulin sensitivity, body composition and linear growth. *Horm Res Paediatr* 2011 **76** 355-366.
2. Polak M, Blair J, Kotnik P, Pournara E, Pedersen BT & Rohrer TR. Early growth hormone treatment start in childhood growth hormone deficiency improves near adult height: analysis from NordiNet(R) International Outcome Study. *Eur J Endocrinol* 2017 **177** 421-429.
