# Supplementary material for: Effectiveness and safety of rhIGF1 therapy in patients with or without Laron syndrome
Source: Eur J Endocrinol. 2020 Nov 17;184(2):267–76. doi: 10.1530/EJE-20-0325 (PMC7849377; doi:10.1530/EJE-20-0325)
Supplement: Table S1: Puberty and previous treatment status of the non-treatment-naïve/prepubertal subgroups (Enrolled population) [file supplementary_table_1.pdf]

Table S1: Puberty and previous treatment status of the non-treatment-naïve/prepubertal subgroups (Enrolled population)

Treatment status	Non-NPP-Laron syndrome (*N* = 17)	Non-NPP-non-Laron syndrome (*N* = 87)
**Pubertal naive**	6%	25%
**Pubertal non-naive**	38%	18%
**GH**	33%	93%
**rhIGF-1**	100%	27%
**Steroids**	-	20%
**GH and rhIGF-1**	33%	20%
**Prepubertal non-naive**	56%	58%
**GH**	33%	94%
**rhIGF-1**	67%	14%
**GH and rhIGF-1**	-	8%
**Steroids**	-	2%

Naïve patients are defined as patients without previous growth promoting therapy except corticosteroids. Patients treated with both GH and IGF-1 are also counted in the separate GH and rhIGF-1 categories.GH, growth hormone; IGF-1; insulin-like growth factor-1; non-NPP, not treatment naïve and/or pubertal.
