# Supplementary material for: Effectiveness and safety of rhIGF1 therapy in patients with or without Laron syndrome
Source: Eur J Endocrinol. 2020 Nov 17;184(2):267–76. doi: 10.1530/EJE-20-0325 (PMC7849377; doi:10.1530/EJE-20-0325)
Supplement: Table S2: Non-treatment naïve/pubertal patient characteristics at baseline, with or without Laron syndrome (Enrolled population) [file supplementary_table_2.pdf]

Table S2: Non-treatment naïve/pubertal patient characteristics at baseline, with or without Laron syndrome (Enrolled population)

	Non-NPP-Laron syndrome (*N* = 17)		Non-NPP-non-Laron syndrome (*N* = 87)		P values[a]
	N[b]		N[b]		
**Female, *n* (%)**<br>95% CI	17	7 (41.2)<br>21.6; 64.0	87	28 (32.2)<br>23.3; 42.6	0.473[c]
**Age at first injection, years**<br>Mean (SD [95% CI])<br>Median (Q1; Q3)	17	 12.78 (3.73 [10.86; 14.70])<br>12.16 (10.72; 15.29)	87	 11.43 (3.58 [10.67; 12.20])<br>12.25 (8.74; 13.93)	0.272[w]
**Height, SDS**<br>Mean (SD [95% CI])<br>Median (Q1; Q3)	15	 –4.63 (1.51 [–5.47; –3.79])<br>–4.93 (–5.89; –3.30)	77	 –3.61 (1.20 [–3.88; –3.34])<br>–3.25 (–4.33; –2.84)	0.011[w]
**Height velocity, cm/year**<br>Mean (SD [95% CI])<br>Median (Q1; Q3)	12	 4.43 (1.23 [3.65; 5.21])<br>4.57 (3.69; 5.18)	52	 4.70 (1.84 [4.19; 5.21])<br>4.53 (3.70; 5.60)	0.884[w]
**Weight, SDS**<br>Mean (SD [95% CI])<br>Median (Q1; Q3)	15	 –2.34 (1.28 [–3.05; –1.63])<br>–2.42 (–3.37; –1.17)	79	 –3.28 (1.54 [–3.62; –2.93])<br>–2.92 (–4.10; –2.34)	0.036[w]
**BMI, SDS**	15		71		<0.001[w]

Mean (SD [95% CI])<br>Median (Q1; Q3)		0.86 (1.56 [–0.00; 1.73])<br>0.81 (–0.45; 1.85)		–0.97 (1.38 [–1.30; –0.65])<br>–0.93 (–1.57; –0.27)	
**Mother's height, cm**<br>Mean (SD [95% CI])<br>Median (Q1; Q3)	16	158.1 (10.2 [152.7; 163.5])<br>158.1 (148.7; 167.5)	76	157.5 (9.2 [155.4; 159.6])<br>158.0 (151.5; 163.6)	-
**Father's height, cm**<br>Mean (SD [95% CI])<br>Median (Q1; Q3)	17	173.2 (5.8 [170.2; 176.2])<br>172.0 (170.0; 178.0)	76	171.4 (8.2 [169.6; 173.3])<br>171.5 (165.3; 177.5)	-
**IGF-1, ng/mL**<br>Mean (SD [95% CI])<br>Median (Q1; Q3)	13	261.41 (205.25 [137.38; 385.44])<br>246.00 (62.00; 462.00)	78	148.74 (144.66 [116.12; 181.35])<br>105.50 (60.00; 171.10)	0.153[w]
**Peak stimulated GH level, ng/mL**<br>Mean (SD [95% CI])<br>Median (Q1; Q3)	9	62.77 (37.09 [34.25; 91.28])<br>55.00 (40.00; 72.90)	52	23.95 (50.31 [9.94; 37.96])<br>15.10 (7.45; 23.25)	<0.001[w]
**Primary diagnosis: SPIGFD[†], *n* (%)**<br>95% CI	17	17 (100)<br>81.6; 100	87	63 (72.4)<br>62.2; 80.7	-
**History of hypoglycaemia, *n* (%)**<br>95% CI	17	5 (29.4)<br>13.3; 53.1	87	5 (5.7)<br>2.5; 12.8	0.010[f]

Data are median Q1; Q3 unless stated otherwise. [†]including Laron syndrome, as reported by the investigator. [a]Non-NPP-Laron syndrome versus Non-NPP-non-Laron syndrome. [b]Number of patients with available data; (c) Chi-square test; (f) Fisher's test; (w) Wilcoxon test. BMI, body mass index; GH, growth

hormone; IGF-1; insulin-like growth factor-1; NPP, treatment-naïve and prepubertal; SD, standard deviation; SDS, standard deviation score; SPIGFD, severe primary IGF-1 deficiency.
