# Supplementary material for: Effectiveness and safety of rhIGF1 therapy in patients with or without Laron syndrome
Source: Eur J Endocrinol. 2020 Nov 17;184(2):267–76. doi: 10.1530/EJE-20-0325 (PMC7849377; doi:10.1530/EJE-20-0325)
Supplement: Table S3. Effect of rhIGF-1 therapy on height SDS in the non-treatment-naïve/prepubertal cohort (Registry population) [file supplementary_table_3.pdf]

Table S3. Effect of rhIGF-1 therapy on height SDS in the non-treatment-naïve/prepubertal cohort (Registry population)

			Change from baseline				Change from baseline	
	N	Mean (SD)	N	Mean (95% CI)	N	Mean (SD)	N	Mean (95% CI)
	Non-NPP-LS (*n* = 17)				Non-NPP-non-LS (*n* = 86)			
1 year	14	–4.27 (1.60)	14	0.19 (–0.10; 0.48)	70	–3.40 (1.32)	62	0.24 (0.13; 0.36)
2 years	14	–4.32 (1.57)	13	0.55 (0.18; 0.92)	47	–3.16 (1.54)	41	0.49 (0.25; 0.72)
3 years	10	–4.68 (1.82)	9	0.34 (–0.07; 0.75)	33	–3.06 (1.58)	27	0.66 (0.27; 1.04)
4 years	9	–4.39 (1.92)	8	0.51 (–0.01; 1.04)	23	–3.35 (1.71)	18	0.75 (0.38; 1.12)
5 years	4	–5.12 (1.36)	4	-	11	–3.47 (2.22)	10	0.61 (0.02; 1.20)

Mean (SD/95% CI) are not reported if the N is <5. CI, confidence interval; N, number of patients with available data at each time point. LS, Laron syndrome; non-NPP, not treatment naïve and/or pubertal; NPP, treatment-naïve and prepubertal; SD, standard deviation; SDS, standard deviation score.
