# Supplementary material for: Effectiveness and safety of rhIGF1 therapy in patients with or without Laron syndrome
Source: Eur J Endocrinol. 2020 Nov 17;184(2):267–76. doi: 10.1530/EJE-20-0325 (PMC7849377; doi:10.1530/EJE-20-0325)
Supplement: Table S4. Effect of rhIGF-1 therapy on height velocity (cm/year; Registry population) [file supplementary_table_4.pdf]

Table S4. Effect of rhIGF-1 therapy on height velocity (cm/year; Registry population)

			**Change from baseline**				**Change from baseline**	
	**N**	**Mean (SD)**	**N**	**Mean (95% CI)**	**N**	**Mean (SD)**	**N**	**Mean (95% CI)**
	**NPP-LS** (*n* = 21)				**NPP-non-LS** (*N* = 114)			
1 year	10	8.25 (2.54)	4	-	87	7.04 (1.96)	52	2.42 (1.75; 3.10)
2 years	7	6.87 (1.54)	2	-	70	6.13 (1.59)	40	1.76 (1.06; 2.47)
3 years	9	5.35 (1.57)	2	-	58	6.08 (1.82)	34	1.36 (0.44; 2.28)
4 years	7	5.35 (0.94)	1	-	38	5.60 (1.69)	19	0.70 (–0.27; 1.67)
5 years	5	5.45 (1.10)	0	-	21	4.87 (1.84)	10	1.12 (–0.07; 2.32)
	**NPP-non-LS–responders** (*n* = 50)				**NPP-non-LS–poor-responders** (*n* = 38)			
1 year	50	8.06 (1.69)	35	3.02 (2.31; 3.74)	37	5.66 (1.36)	17	1.18 (–0.19; 2.55)
2 years	38	6.18 (1.50)	27	1.49 (0.78; 2.19)	29	6.05 (1.79)	13	2.34 (0.60; 4.08)
3 years	31	5.95 (1.59)	23	0.77 (–0.16; 1.70)	20	6.50 (2.22)	9	2.43 (–0.30; 5.15)
4 years	19	5.56 (1.19)	14	0.46 (–0.45; 1.36)	11	5.37 (1.88)	4	-
5 years	14	5.06 (2.04)	8	1.01 (–0.52; 2.55)	3	4.64 (1.42)	2	-
	**Non-NPP-LS**				**Non-NPP-non-LS**			

	(*n* = 17)				(*n* = 86)			
1 year	13	5.50 (2.88)	10	0.38 (–1.32; 2.09)	59	6.48 (2.37)	41	1.73 (0.86; 2.59)
2 years	11	4.90 (2.38)	9	0.04 (–1.40; 1.47)	35	6.00 (2.40)	22	0.68 (–0.43; 1.79)
3 years	10	3.42 (3.21)	7	–2.08 (–5.33; 1.17)	25	5.72 (2.17)	13	0.99 (–0.42; 2.41)
4 years	7	4.55 (2.45)	4	-	16	4.77 (2.11)	7	0.18 (–1.70; 2.05)
5 years	3	4.34 (1.07)	2	-	9	4.35 (2.09)	5	–0.65 (–2.90; 1.60)

Mean (SD/95% CI) are not reported if the N is <5. No statistical analysis was conducted for height velocity. Responders were defined as patients with change in height SDS in year 1 of ≥0.3. Poor-responders were defined as patients with change in height SDS in year 1 of <0.3. CI, confidence interval; N, number of patients with available data at each time point. LS, Laron syndrome; non-NPP, not treatment naïve and/or pubertal; NPP, treatment-naïve and prepubertal; SD, standard deviation; SDS, standard deviation score.
