# Supplementary material for: Effectiveness and safety of rhIGF1 therapy in patients with or without Laron syndrome
Source: Eur J Endocrinol. 2020 Nov 17;184(2):267–76. doi: 10.1530/EJE-20-0325 (PMC7849377; doi:10.1530/EJE-20-0325)
Supplement: Table S5. Effect of rhIGF-1 therapy on BMI SDS (Registry population) [file supplementary_table_5.pdf]

Table S5. Effect of rhIGF-1 therapy on BMI SDS (Registry population)

	Change from baseline		Change from baseline		P value[a]
	N	Mean (95% CI)	N	Mean (95% CI)	
	NPP-LS (*n* = 21)		NPP-non-LS (*N* = 114)		
1 year	10	0.44 (0; 1.23)	77	0.11 (0; 0.24)	0.744
2 years	10	0.55 (0; 1.53)	68	0.21 (0.08; 0.65)	0.828
3 years	9	0.78 (0; 1.89)	56	0.29 (0.11; 0.46)	0.258
4 years	8	0.99 (0; 2.16)	42	0.20 (0; 0.42)	0.142
5 years	8	1.16 (0.19; 2.14)	25	0.30 (0; 0.66)	0.062
	NPP-non-LS–responders (*n* = 50)		NPP-non-LS–poor-responders (*n* = 38)		P value[b]
1 year	44	0.10 (0; 0.31)	33	0.12 (0; 0.27)	0.930
2 years	35	0.21 (0.01; 0.41)	25	0.19 (0.01; 0.36)	0.862
3 years	30	0.21 (0; 0.47)	20	0.34 (0.12; 0.56)	0.467
4 years	23	0.11 (0; 0.44)	11	0.36 (0; 0.77)	0.357
5 years	17	0.38 (0; 0.88)	5	0.29 (0.06; 0.52)	0.875
	Non-NPP-LS (*n* = 17)		Non-NPP-non-LS (*n* = 86)		
1 year	14	0.01 (0.01; 0.32)	56	0.35 (0.19; 0.52)	-
2 years	13	–0.00 (0; 0.48)	37	0.45 (0.23; 0.66)	-
3 years	9	0.06 (0; 0.49)	24	0.51 (0.26; 0.76)	-
4 years	8	0.35 (0; 1.27)	15	0.56(0.25; 0.88)	-
5 years	4	0.17 (0; 1.27)	10	0.70 (0.08; 1.32)	-

[a]NPP-non-Laron syndrome versus NPP-Laron syndrome mean BMI SDS. [b]NPP-non-Laron syndrome responders versus NPP-non-Laron syndrome-poor responders mean BMI SDS. Responders were defined as patients with change in height SDS in year 1 of ≥0.3. Poor-responders were defined as patients with change in height SDS in year 1 of <0.3. CI, confidence interval; N, number of patients with available data at each time point. LS, Laron syndrome; non-NPP, not treatment naïve and/or pubertal; NPP, treatment-naïve and prepubertal; SDS, standard deviation score.
