# Supplementary material for: Effectiveness and safety of rhIGF1 therapy in patients with or without Laron syndrome
Source: Eur J Endocrinol. 2020 Nov 17;184(2):267–76. doi: 10.1530/EJE-20-0325 (PMC7849377; doi:10.1530/EJE-20-0325)
Supplement: Table S6. Effect of rhIGF-1 therapy on weight SDS (Registry population) [file supplementary_table_6.pdf]

Table S6. Effect of rhIGF-1 therapy on weight SDS (Registry population)

	Change from baseline		Change from baseline		P value[a]
	**N**	**Mean (95% CI)**	**N**	**Mean (95% CI)**	
	**NPP-LS** (*n* = 21)		**NPP-non-LS** (*N* = 114)		
1 year	11	0.81 (0.01; 1.61)	88	0.37 (0.26; 0.49)	0.298
2 years	11	1.19 (0; 2.40)	77	0.70 (0.54; 0.87)	0.419
3 years	9	1.76 (0.12; 3.39)	62	0.89 (0.70; 1.07)	0.099
4 years	8	2.01 (0.55; 3.48)	46	1.05 (0.78; 1.32)	0.022
5 years	8	2.02 (0.49; 3.56)	29	1.24 (0.88; 1.61)	0.109
	**NPP-non-LS–responders** (*n* = 50)		**NPP-non-LS–poor-responders** (*n* = 38)		**P value[b]**
1 year	50	0.55 (0.39; 0.70)	37	0.13 (0; 0.28)	<0.001
2 years	40	0.96 (0.77; 1.15)	28	0.24 (0.04; 0.45)	<0.001
3 years	33	1.05 (0.79; 1.31)	22	0.57 (0.27; 0.88)	0.017
4 years	25	1.17 (0.76; 1.58)	12	0.74 (0.18; 1.30)	0.189
5 years	20	1.42 (0.95; 1.88)	5	0.41 (0; 1.33)	0.038
	**Non-NPP-LS**		**Non-NPP non-LS**		

	(*n* = 17)		(*n* = 86)		
1 year	14	0.27 (0; 0.58)	63	0.42 (0.27; 0.58)	-
2 years	13	0.50 (0.14; 0.85)	41	0.76 (0.50; 1.03)	-
3 years	9	0.51 (0.22; 0.79)	27	1.02 (0.63; 1.42)	-
4 years	8	0.97 (0.05; 1.89)	18	1.28 (0.94; 1.61)	-
5 years	4	1.10 (0.31; 1.89)	10	1.43 (1.05; 1.82)	-

[a]NPP-non-Laron syndrome versus NPP-Laron syndrome mean weight SDS. [b]NPP-non-Laron syndrome responders versus NPP-non-Laron syndrome-poor responders mean weight SDS. Responders were defined as patients with change in height SDS in year 1 of ≥0.3. Poor-responders were defined as patients with change in height SDS in year 1 of <0.3. CI, confidence interval; N, number of patients with available data at each time point. LS, Laron syndrome; non-NPP, not treatment naïve and/or pubertal; NPP, treatment-naïve and prepubertal; SDS, standard deviation score.
